# 5P Strategies for Management of Multiple Endocrine Neoplasia Type 2: A Paradigm of Precision Medicine

**DOI:** 10.3389/fendo.2020.543246

**Published:** 2020-09-18

**Authors:** Shu-Yuan Li, Yi-Qiang Ding, You-Liang Si, Mu-Jin Ye, Chen-Ming Xu, Xiao-Ping Qi

**Affiliations:** ^1^International Peace Maternity and Child Health Hospital, School of Medicine, Shanghai Jiao Tong University, Shanghai, China; ^2^Department of Oncologic and Urologic Surgery, The 903rd PLA Hospital, Wenzhou Medical University, Hangzhou, China

**Keywords:** 5P strategies, multiple endocrine neoplasia type 2, medullary thyroid carcinoma, pheochromocytoma, RET proto-oncogene, precision medicine

## Abstract

Multiple endocrine neoplasia type 2 (MEN2) is a neuroendocrine cancer syndrome characterized by medullary thyroid carcinoma, in combination or not with pheochromocytoma, hyperparathyroidism, and extra-endocrine features. MEN2 syndrome includes two clinically distinct forms subtyped as MEN2A and MEN2B. Nearly all MEN2 cases are caused by germline mutations of the *RET* proto-oncogene. In this review, we propose “5P” strategies for management of MEN2: prevention, prediction, personalization, psychological support, and participation, which could effectively improve clinical outcomes of patients. Based on *RET* mutations, MEN2 could be *prevented* through prenatal diagnosis or preimplantation genetic testing. Identification of pathogenic mutations in *RET* can enable early diagnosis of MEN2. Combining *RET* mutation testing with measurement of serum calcitonin, plasma or urinary metanephrine/normetanephrine, and serum parathyroid hormone levels could allow risk stratification and accurately *prediction* of MEN2 progression, thus facilitating implementation of *personalized* precision treatments to increase disease-free survival and overall survival. Furthermore, increased awareness of MEN2 is needed, which requires *participation* of physicians, patients, family members, and related organizations. *Psychological* support is also important for patients with MEN2 to promote comprehensive management of MEN2 symptoms. The “5P” strategies for management of MEN2 represent a typical clinical example of precision medicine. These strategies could effectively improve the health of MEN2 patient, and avoid adverse outcomes, including death and major morbidity, from MEN2.

## Introduction

Multiple endocrine neoplasia type 2 (MEN2) is a neuroendocrine cancer syndrome characterized by medullary thyroid carcinoma (MTC), in combination or not with pheochromocytoma (PHEO), hyperparathyroidism (HPTH), and extra-endocrine features (1). MEN2 has two clinically distinct forms subtyped as MEN2A (OMIM# 171400; ~95% of MEN2) and MEN2B (OMIM# 162300; ~5%) (2, 3). MEN2A can be classified into 4 variants, including classical MEN2A (~60–70% of MEN2A cases), MEN2A with cutaneous lichen amyloidosis (CLA) (~9%), MEN2A with Hirschsprung disease (HD) (~7%), and familial MTC (FMTC; OMIM #155240) (~15%) (2). MEN2B is characterized by highly aggressive MTC, PHEO, and extra-endocrine manifestations, including mucosal neuromas, ocular signs, marfanoid habitus and other musculoskeletal features, and constipation, diffuse ganglioneuromatosis of the gastrointestinal tract (4, 5). All MEN2 subtypes are inherited in an autosomal dominant pattern with high penetrance. Nearly all MEN2 cases are caused by germline gain of function mutations of the REarranged during Transfection (*RET*) proto-oncogene, with the exception of two families having germline mutations in *ESR2* or *MET* gene that are predisposed to MTC (2, 6, 7). Over the last two decades, identification of *RET* mutations as the cause of MEN2 significantly changed MEN2 disease management, including disease prevention, diagnosis, risk prediction, and treatment of MEN2-specific tumors; together these approaches represent a paradigm of precision medicine (2). In this review, we summarize the genetic characteristics, molecular diagnosis and management of MEN2, with particular focus on “5P” strategies: prevention, prediction, personalization, psychological support, and participation, which could effectively improve clinical outcomes for patients with MEN2-specific tumors.

## Genotype-Phenotype Correlation in MEN2

**Figure 1:**
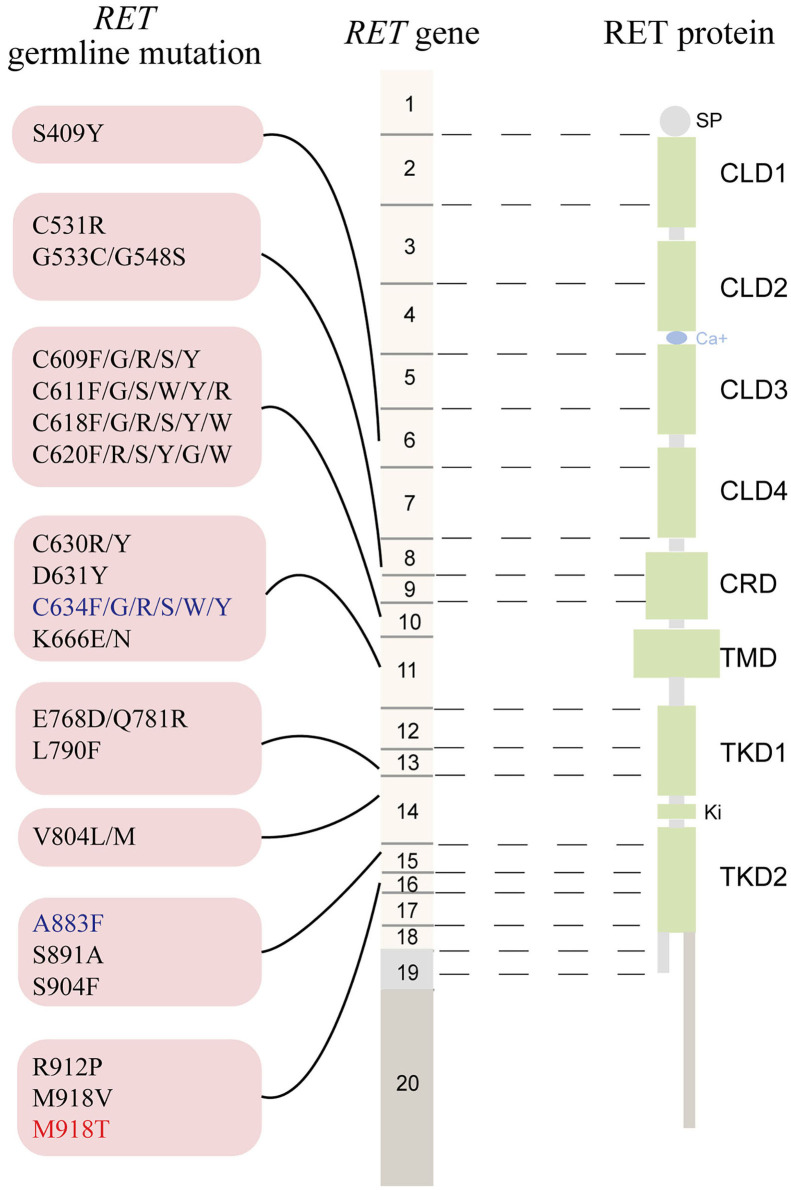
The schematic representation the *RET* gene (middle), the RET protein (right), and common *RET* germline mutations (left). Only pathogenic mutations reported in two or more families or unrelated cases with MEN2 in the MEN2 Database (www.arup.utah.edu/database/men2/MEN2_display.php (accessed May, 2019) and one novel mutation S409Y identified by our group recently were presented. Alternative splicing involving exon 19, intron 19, and exon 20 produces two main alternatively spliced forms of the RET protein, RET9 (short isoform, 1072 residues) and RET 51 (long isoform, 1114 residues), are indicated in light gray and light brown, respectively. The mutations shown in red, blue, and black represent the highest, high, moderate risk of aggressive medullary thyroid carcinoma according to the revised American Thyroid Association guideline for the management of medullary thyroid carcinoma. SP, signal peptide; CLD, cadherin-like domain; CRD, cysteine-rich domain; TMD, transmembrane domain; TKD, tyrosine kinase domain.

The *RET* gene, located on chromosome 10q11.2, contains 20 exons and encodes a tyrosine kinase (TK) receptor (https://www.ncbi.nlm.nih.gov/gene/5979). To date, ~90 pathogenic variants are associated with MEN2 (www.arup.utah.edu/database/MEN2/MEN2_welcome.php) (Figure 1). Most *RET* mutations in MEN2 are heterozygous mutations, although homozygous mutations, double/multiple mutations, duplications, insertions, or deletions also occur (8, 9). The mutation hotspots mainly concentrate in exons 8, 10, 11, and 13–16 of *RET* (2).

Approximately 95% of MEN2A cases have germline missense mutations in exons 10 (codons 609, 611, 618, or 620) and 11 (codon 634), which encode the cysteine-rich domain (CRD) of the *RET* extracellular domain (10). Although the relative prevalence of the *RET* C634 mutation in MEN2 cases has decreased over time as we identify more less severe mutations and families with less penetrant disease, it remains the most common mutation and is found in ~40% of patients with MEN2A (11). According to the revised American Thyroid Association Guidelines for the management of MTC (hereafter referred to as “ATA-2015”), patients carrying the *RET* C634 mutation are in the “high risk” (ATA-H) category for developing hereditary aggressive MTC, whereas patients with *RET* mutations in codons 609, 611, 618, or 620 are in the “moderate risk” (ATA-MOD) category (Table 1, Figure 1) (2). The *RET* C634 mutation is also associated with higher penetrance of PHEO (~50%) and HPTH (20–30%), compared to mutations in codons 609 (26%; 5%), 611 (10%; 2%), 618 (23%; 3%), or 620 (13%; 2%) (2, 12). In addition, nearly all patients with MEN2A and CLA carry the *RET* C634 mutation (96.4%), although one patient with a V804M mutation (1.8%) and another with a C611Y mutation (1.8%) have been reported (13–16). The prevalence of MTC, PHEO, and HPTH in individuals with MEN2-related CLA was ~96.2, 50.9, and 13.2%, respectively (Table 2). Individuals with MEN2A and HD have mutations in *RET* involving codons in exon 10, including C620 (28–50%, the most common), C618 (27–30%), C611 (5–40%), and C609 (15–17%) (2, 29). It should be noted that CLA and HD in MEN2 might represent earlier and “premonitory” symptoms.

**Table 1:** The management of MEN2 patients in the ATA guidelines for the MTC (ATA-2015).

**ATA-2015**	**M918T**	**C634/a883f**	**Codon mutations other than M918T, C634, and A883F**
MTC risk category^a^	HST	H	MOD
MEN2 subtype	MEN2B	MEN2A/MEN2B	MEN2A
Timing of PTT	The first year or the first months of life	At or before 5 years of age based on serum Ctn level	When serum Ctn level rise, or in childhood based on parents' wishes
Screening for PHEO	Begin at 11 years of age (annually)	Begin at 11 years of age (annually)	Begin at 16 years of age
Screening for HPTH	–	Begin at 11 years of age	Begin at 16 years of age

a *Risk of aggressive MTC: MOD, moderate; H, high; HST, highest*.

*ATA, American Thyroid Association; MTC, medullary thyroid carcinoma; PTT, prophylactic thyroidectomy; PHEO, pheochromocytoma; HPTH, hyperparathyroidism; ASAP, as soon as possible; Ctn, calcitonin; MEN2A, multiple endocrine neoplasia type 2A; MEN2B, multiple endocrine neoplasia type 2B; FMTC, familial medullary thyroid carcinoma*.

**Table 2:** Clinical data of specific *RET* mutation and disease phenotype for CLA and MEN2A.

***RET***	**Families**,	***RET* carriers**,	**CLA**			**MTC/PHEO/HPT in MEN2A**			**References**
**Mutation**	***n* (%)**	***n* (%)**				**patients with CLA**, ***n*** **(%)**			
			***n* (%)**	**Female, *n* (%)**	**ADC, year**	**MTC**	**PHEO**	**HPT**	
C611Y	1 (4.5)	17 (12.0)	1 (5.9)	1 (100)	40	1	1	0	(15)
C634F	1 (4.5)	3 (2.1)	1 (33.3)	1 (100)	57	1	0	0	(16)
C634G	3 (13.6)	7 (4.9)	5 (71.4)	3 (60)	44/53/54/54/56	5	3	0	(16, 17)
C634R	7 (31.8)	42 (29.6)	15 (35.7)	13 (86.7)	9/14/15/18/21/24/25/47/56^a^	11^a^	6^a^	2^a^	(14, 18–21)
C634S	1(4.5)	1 (0.7)	1 (100)	0 (0)	36	1	1	0	Unpublished
C634W	2 (9.0)	11 (7.7)	6 (54.5)	5 (83.3)	18/20/27/28/46/60	6	4	2	(14, 22)
C634Y	5 (22.7)	55 (38.7)	24 (45.3)^a^	17(70.8)	5/5/10/10/11/13/ 14/17/25/30/34/40/45/52^a^	22	12	3	(20, 23–27)
C634^a^	1 (4.5)	3 (2.1)	3 (100)	3 (100)	14/39/40	3	0	0	(28)
V804M	1 (4.5)	3 (2.1)	1 (33.3)	1 (100)	50	1	0	0	(13)
Total	22 (100)	142 (100)	57 (40.7)^a^	44 (77.2)^a^	31.1^a^	51/53 (96.2)^a^	27/53 (50.9)^a^	7/53 (13.2)^a^	

a *Analyzed based on available data*.

*CLA, cutaneous lichen amyloidosis; MEN2A, multiple endocrine neoplasia type 2A; ADC, age at diagnosis of CLA; MTC, medullary thyroid carcinoma; PHEO, pheochromocytoma; HPT, hyperparathyroidism*.

Approximately 95% of patients with MEN2B have the germline mutation M918T in exon 16 and fewer than 5% have mutation A883F in exon 15 (Table 1, Figure 1) (2). Double *RET* mutations in tandem involving V804M in exon 14 and either Y806C, S904C, E805K, or Q781R are present in a rare group of patients with MEN2B (2, 9). Around 90% of the mutations in patients with MEN2B occur *de novo* (M918T, 93%; A883F, 45%) and are of paternal origin (5, 30, 31). Patients with the *RET* M918T mutation are in the “highest risk” (ATA-HST) category for developing aggressive MTC, whereas patients with A883F belong to ATA-H category; Of these, ~50% of MEN2B patients develop PHEO (2, 4, 32). A recent study identified the *RET* M918V mutation in eight kindreds, none of whom presented clinical features of MEN2B. The *RET* M918V mutation was also identified in a 69-year-old female patient presenting with left single MTC in our center, similar to previous studies (33, 34). Moreover, patients with double tandem mutations present with atypical MEN2B, characterized by MTC with a relatively late age of onset and varying aggressiveness, and none had PHEO. Nonetheless, all individuals with MEN2B present a unique physical appearance characterized by extra-endocrine manifestation MEN2B patients may also have clinically insignificant parathyroid adenoma/proliferation (2, 4, 35).

## 5P Strategies for Management of MEN2

**Figure 2:**
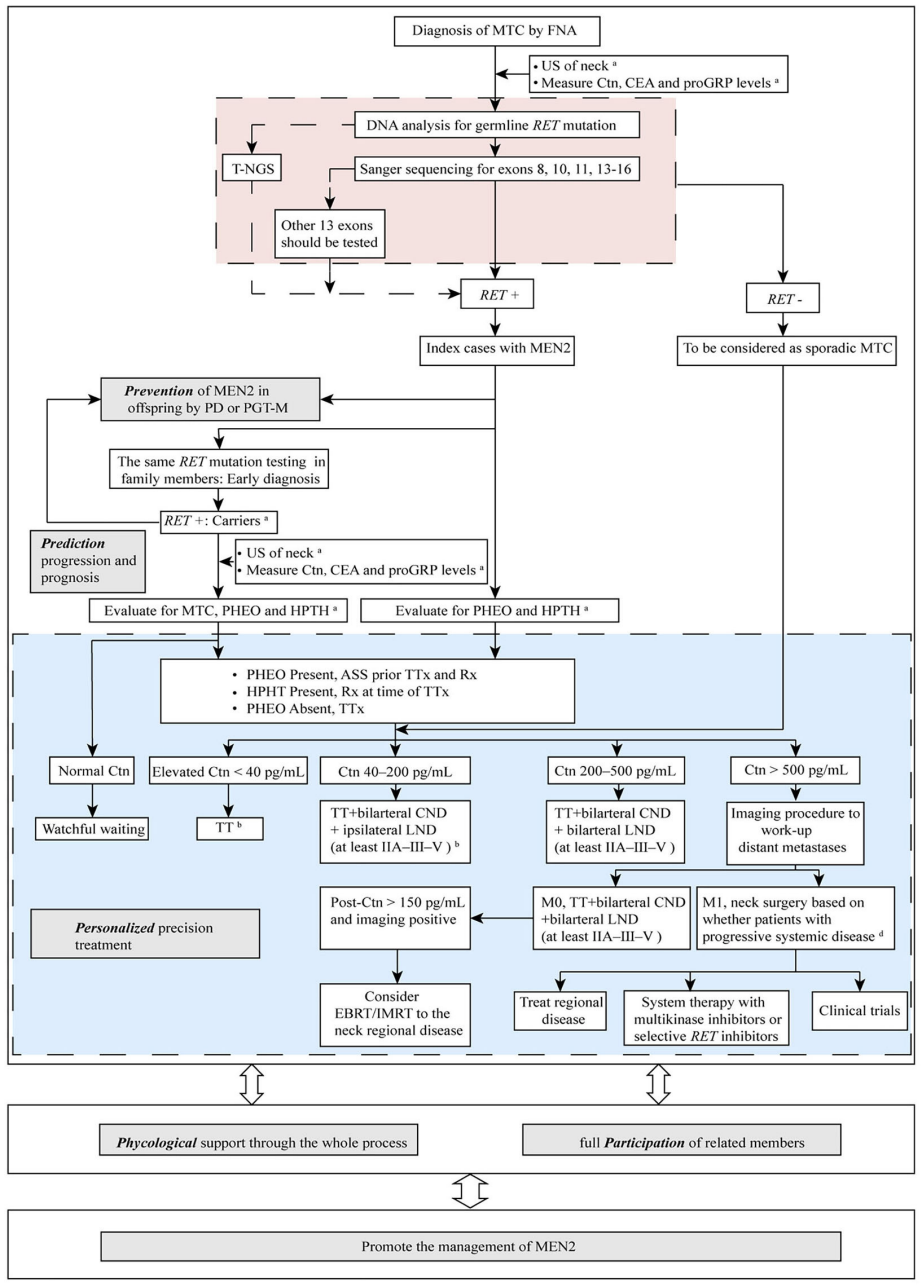
The “5P” strategies for the management of MEN2. ^a^See Table 1 and text details for timing of prophylactic TT, begin screening for PHEO and HPTH; ^b^ESMO Clinical Practice Guidelines on management of thyroid cancer (2019) recommended TT should be performed when elevated Ctn < 20 pg/mL. If MTC is discovered after lobectomy, consider completion thyroidectomy unless postoperative Ctn is undetectable, neck US normal and no germline *RET* mutation is found. When Ctn 20–50 pg/mL, TT ± bilarteral CND should be performed (36); ^c^Decision for surgery may be based on tumor burden in the neck as compared with tumor burden outside the neck in distant metastases (M1). MTC, medullary thyroid carcinoma; FNA, fine needle aspiration; US, ultrasound; Ctn, calcitonin; CEA, carcinoembryonic antigen; proGRP, pro-gastrin-releasing peptide; *RET*, REarranged during Transfection; T-NGS, targeted next generation sequencing; MEN2, multiple endocrine neoplasia type 2; PD, prenatal diagnosis; PGT-M, preimplantation genetic testing for monogenic disorders; PHEO, pheochromocytoma; HPTH, hyperparathyroidism; ASS, adrenal-sparing surgery; TT, total thyroidectomy; TTx, TT with variable extent of neck dissection; Rx, medication/surgery; CND, central neck dissection; LND, lymph node dissection; M1, metastatic MTC; EBRT, external beam radiotherapy; IMRT, intensity-modulated radiation therapy.

Strategies to manage MEN2 can be termed “5P” to describe efforts to *prevent, predict, personalize*, offer *psychological* support, *and participate*, which together can be used to improve clinical outcomes for patients with MEN2. In the subsequent sections we describe these individual strategies (Figure 2).

### Prevention of MEN2

Offspring of individuals with MEN2 have a 50% chance of inheriting the pathogenic variant and developing the disease. Thus, reproductive options, including prenatal diagnosis (PD) and preimplantation genetic testing for monogenic disorders (PGT-M), can be provided to these patients to prevent MEN2 transmission (2). Based on the identified *RET* mutation in the family, PD and PGT-M can be performed by chorionic villus sampling or amniocentesis, or by cleavage stage biopsy or blastocyst biopsy, respectively. PGT-M is an *in vitro* fertilization technique with the advantage of avoiding the need for invasive prenatal diagnosis, thereby circumventing arbitrary decisions regarding pregnancy termination (37). Compared to timely prophylactic thyroidectomy (PTT, discussed below), PGT-M is a primary prevention strategy which can avoid the transmission of diseases and families at risk from the source (38). However, the potential risk (such as implantation failure, multiple pregnancies, premature birth, etc.), and the high cost (i.e., more than $4,000 for one PGT cycle and not covered by health insurance in China) have hindered the widespread use of PGT in MEN2. Moreover, it should be noted that ethical debatable are exist for the usage of PGT in this adult-onset disease with alternative management methods in some countries (2, 39). As demonstrated in the ATA-2015, the clinicians should make patients aware of both the benefits and the potential risks of these technologies, and genetic counseling about these reproductive options should be considered for all patients carrying the *RET* mutations who are of reproductive age, particularly those having *RET* mutations in codons 634 and 918 (2). A simple and efficient method that involves targeted and capture-based next generation sequencing (T-NGS) for identification of informative markers including the entire *RET* coding region and 1 Mb range on each side of the gene together with Sanger sequencing has been established by our group for PGT-M of MEN2 (38). Using this method, one healthy baby whose father had MEN2A with the *RET* C634Y mutation was born without the inheritance of the mutation. However, PD and PGT-M, which are both invasive procedures, are not suitable for prevention of cases caused by *de nov*o *RET* mutation, as occurs for 90% of MEN2B index cases with a negative family history. Nonetheless, the PD/PGT-M can also be applied to MEN2B patients (parents) who wish to have offspring. Non-invasive prenatal screening (NIPS) of MEN2 using cell-free fetal DNA from maternal plasma was successfully used for couples in which only the father was *RET* mutation carrier by Macher et al. in the year of 2012 (40). Recently, NIPS for many monogenic diseases associated with mutations of maternal or paternal origin or occurred *de novo* using NGS or Droplet Digital PCR has been reported (41–43). These techniques could play important roles in pregnancy management and prevention of human monogenic disorders, such as MEN2, especially MEN2B.

### Prediction of MEN2 Progression and Prognosis

Establishing a diagnosis and predicting the progression of MEN2 at an early age is essential to improve the likelihood of good outcomes. Measurement of bCtn levels was previously the most important screening method for subclinical or early stage MTC (44). MTC had not developed when bCtn levels were within the reference range, and patients with normal or undetectable bCtn levels after initial thyroidectomy are considered “biochemically cured” with a 10-year survival rate of 97.7 or 100%, respectively (2, 45). Although bCtn testing is useful, the relatively high incidence of false positives (5–10%) can lead to unnecessary surgery (46). In contrast, bCtn levels are undetectable in 0.83% of patients (7/839) with advanced MTC (47). Moreover, the reference ranges of bCtn and stimulated Ctn (sCtn) levels are age- and gender-dependent, and may vary depending on the commercial assays used (48). Following the discovery of *RET* mutations as the cause of MEN2, the management of MEN2 has changed profoundly. *RET* mutation testing has superior performance over b/sCtn measurement in terms of providing clearer delineation of risks for patients with MEN2 and their family members, as well as having lower false negative and positive rates (49). The identification of *RET* pathogenic mutations can enable the accurate diagnosis of MEN2 before the onset of clinical symptoms, avoid the need for repeated biochemical screening for individuals who did not inherit the mutation and concentrate resources on those who actually are mutation carriers (49, 50). *RET* mutation testing can also facilitate early diagnosis and treatment of MEN2-related diseases (e.g., CLA, HD) (15, 16). In addition, 1–7% of patients with presumed sporadic MTC actually have MEN2 or hereditary MTC. Genetic testing is beneficial for correcting an original diagnosis of “sporadic MTC” for these groups of patients and for the treatment and management of their relatives (2, 33). For MEN2B, which is most frequently due to *de novo RET* mutations, performance of large-scale clinical screening via genetic testing is impractical, which makes the timely diagnosis of MEN2B challenging. With the improved acknowledge of MEN2B, *RET* mutation testing together with consideration of early features of MEN2B-related non-endocrine disorders is critical to minimize delays in diagnosis and to improve outcomes of MEN2B (2, 4, 5). Germline *RET* mutation analysis currently plays a pivotal role in the clinical management of MTC, which should be offered to the following individuals (2, 51): (1) all MEN2 patients; (2) first-degree relatives of hereditary MTC patients; (3) CLA patients; (4) HD patients; (5) parents whose infants or young children have the classic phenotype of MEN2B; and (6) “sporadic MTC” patients (2). With respect to *RET* mutation screening strategies, mutations in exons 8, 10, 11, and 13–16 should be first tested for MEN2A patients, whereas initial testing for the mutation M918T in exon 16 and A883F in exon 15 (when negative for M918T) should be performed for MEN2B cases. The entire *RET* coding region should be sequenced when the initial test is negative or if the patient's phenotype is inconsistent with the *RET* mutation that was initially identified (2). Based on these strategies, we identified 23 *RET* mutations in 73 MEN2 families, most of which are located in hotspot mutation regions (52–59). Recently, our group used T-NGS to identify a novel mutation outside the hotspot mutation regions (S409Y in exon 6 of *RET*) in four families (33). These results demonstrate that T-NGS is an accurate, rapid, and practical sequencing strategy for mutation screening of *RET* in MEN2 cases. As the cost of NGS decreases and ethical and other standards are developed, resequencing of the entire *RET* region using T-NGS will probably supersede existing step-by-step detection strategies to become a routine method for the mutation screening of MEN2 cases (16, 53, 59, 60).

Almost all patients with MEN2-related MTC/PHEO present with high levels of bCtn (>99%) and free plasma or 24-h urinary metanephrine/normetanephrine (MN/NMN) (2, 47, 61). Thus, integrating *RET* mutations testing with measurement of bCtn/MN/NMN levels can be used to accurately predict disease progression and prognosis. The levels of bCtn were shown to be closely correlated with MTC burden, with initially bCtn levels >40 pg/mL be considered local nodal metastases, >500 pg/mL be considered distant metastases, and >3,000 pg/mL be considered wide-spread metastases and an inability to cure the patient despite aggressive surgery (2, 62). In addition to bCtn, serum carcinoembryonic antigen (CEA) levels are also associated with MTC risk, with CEA levels >30 ng/mL suggesting that the disease is unlikely to be cured with surgery, and CEA levels >100 ng/mL signifying extensive lymph node and distant metastases (63). Recently, pro-gastrin-releasing peptide (proGRP) was suggested to be a potential marker of MTC and thus the combination of proGRP, bCtn, and CEA levels may be additionally helpful in the evaluation of MTC stage (64, 65). Ide et al. reported three MTC patients with normal proGRP and N0 stage, but presented high levels of bCtn (110, 957, and 1,410 pg/mL, respectively) (65). Moreover, one patient treated at our center who had the mutation C634R presented with T2aN0M0 stage MTC and initially markedly elevated bCtn (3600.80 pg/mL) and slightly elevated CEA (18.20 ng/mL), whereas the proGRP levels were consistently within the reference range. These results might imply that MTC patients with normal proGRP levels, despite elevated bCtn levels, had no regional lymph node MTC metastasis. In addition, negative MN/NMN measurements should be considered for ruling out MEN2-related PHEO (66). Meanwhile, patients with higher MN/NMN levels may be more prone to postoperative hypertension, although this condition was rarely seen (67).

### Personalized Precision Treatment of MEN2

PTT, the early removal of the bilateral thyroid gland in individuals/children who have inherited a mutated *RET* allele before MTC develops or while it is clinically unapparent and confined to the gland, is currently the most effective method for preventing or curing MEN2-related MTC. The timing of thyroidectomy is mainly based on the perceived clinical behavior of the specific *RET* mutation causing MEN2-related MTC (Table 1) (2). Given the substantial variability in the age at which MTC develops, even among individuals with the same *RET* mutation in the same family, it is generally accepted that the timing of PTT should be determined based on results for integrated *RET* testing and b/sCtn levels (1, 2, 45, 68) (Table 1, Figure 2). For MEN2B individuals with ATA-HST mutation (*RET* M918T), PTT should be performed in the first year of life, which was in accordance with findings by recent study conducted by Castinetti et al. (including our group) demonstrating that PTT performed when patients were 1 year old or younger was associated with a high probability of cure (20 patients) (4). Early clinical recognition of extra-endocrine features becomes paramount in improving surgical cure rates of MEN2B patients (4, 5). For individuals with an ATA-H mutation, the ATA-2015 recommended that PTT should be performed before age 5. For individuals with the ATA-MOD mutation, PTT should be performed when the bCtn/CEA levels are elevated or during childhood based on the parents' wishes. The results of our studies also demonstrated that patients with an ATA-MOD mutation and normal bCtn levels should be actively followed up and monitored, whereas PTT should be performed as early as possible for patients with ATA-H or ATA-HST mutation and presence of CCH/MTC and elevated bCtn levels after the patients turned 5 years-old (53, 56, 57). Annual physical examination, cervical US, and measurement of bCtn/CEA levels should begin at 5 years of age for individuals with ATA-H or ATA-MOD mutations who do not undergo PTT (2).

Based on the benefits of PTT, this approach has been applied to the majority of patients who diagnosed in early age before MTC has developed or are members of well-known *RET*-defined MEN2 families. Over the past 20 years, the prognosis of these patients has greatly improved (69–71). The disease-specific survival rate at 20 years after PTT were 89.9, 93.2, and 54.6% for patients with MOD, H, and HST *RET* mutation, respectively (71). The preferred operation is TT with or without central neck dissection (CND; i.e., level VI dissection). TT without CND is applicable for patients with ATA-MOD~H mutations and bCtn <40 pg/mL (2, 70). When MTC remains confined to the thyroid gland, we and Elisei et al. showed that prophylactic CND could be avoided when bCtn levels were <71.4 and <60 pg/mL, respectively (45, 56). However, ipsilateral lateral neck dissection (ipsilateral levels IIA-III–V and bilateral CND) should be performed if US or physical exam detects lymphadenopathy in the lateral neck, involvement of CND, or primary MTC is >1 cm. Extensive neck dissection (bilateral levels II–III–V and VI) should be considered when patients have bilateral MTCs or extensive lymph node MTC metastasis on the ipsilateral side, and initial bCtn levels >200 pg/mL (Figure 2) (2, 36, 62, 72).

It should be noted, however, that disruption of calcium homeostasis is the most common complication after TT, with a transient hypocalcaemia (within 24 h) reported in over 25% and “permanent” hypoparathyroidism (>6 months) in up to 12% of all adult patients (73). In line with the concept of greater vulnerability of infants/children (≤18 years), de Jong et al. reported 63 children (59.4%) developed transient, and permanent hypoparathyroidism occurred in 23 children (21.7%) (74). Furthermore, compared to patients under TT without CND, the incidence of complications, including transient and permanent hypoparathyroidism or recurrent laryngeal nerve palsy, in patients after TT with CND were higher with longer hospital stay (Table 3) (70, 75, 76). The postoperative hypoparathyroidism was also more prevalent in children younger than 3 years at surgery in particular (11, 75, 77). However, there were also studies reported that age at surgery in children was not associated with these complications (70, 74, 78). Several strategies, including meticulous intraoperative identification to reduce local damage using optical magnification or nerve-monitoring devices, preserve parathyroid glands *in situ*, and transfer patients to higher-volume surgeons, may be useful in improving the outcomes (11, 74, 79). The MEN2 risk and its related postoperative complications also demonstrated the potential benefits of PD and PGT-M, as discussed before.

**Table 3:** Complications after total thyroidectomy with CND and without CND in children (≤18 years).

**References**	**Patients, *n***	**Complication after TT**					
		**TT without CND**			**TT with CND**		
		**Patients, n**	**Transient/permanent RLNP, *n* (%)**	**Transient/permanent HOP, *n* (%)**	**Patients, n**	**Transient/Permanent RLNP, *n* (%)**	**Transient/permanent HOP, *n* (%)**
Kluijfhout et al. (75)	44	44	2 (4.5)/1 (2.3)	12 (27.3)/9 (20.5)	/	/	/
Prete et al. (70)	79	54	0/0	9 (16.7)/6 (11.1)	25	0/0	13 (52.0)/9 (36.0)
Machens et al. (76)	167	109	0/0	19 (17.4)/0	58	3 (5.2)/0	12 (20.7)/0
Total	290	207	2 (1.0)/1 (0.5)	40 (19.3)/15 (7.2)	83	3 (3.6)/0	25 (30.1)/9 (10.8)

*n, number; TT, Total thyroidectomy; CND, Central neck dissection; RLNP, Recurrent laryngeal nerve palsy; HOP, hypoparathyroidism*.

In addition, after PTT or TT, long term patient follow-up is indicated. The doubling time of bCtn and CEA are powerful prognostic indicators of MTC progression, especially the bCtn doubling time (80). Levels of bCtn and CEA should be measured at 3 months postoperatively, and then every 6 months for 1 year, and then yearly if they are undetectable or within the normal range (2). Physical examinations and neck US should also be performed every 6–12 months. When bCtn levels exceed 150 pg/mL, imaging procedures, including neck US, chest CT, contrast-enhanced CT/MRI of the liver, bone scintigraphy and MRI of the pelvis and axial skeleton, and/or PET/CT should be performed to detect persistent, residual, or metastatic MTC (2, 81, 82). For patients with biochemical recurrence with negative imaging, a watchful waiting approach could be applied. Treatment and management options for patients with clinical recurrent MTC after surgery or metastatic MTC that is not amenable to reoperative (palliative) surgery are chemotherapy, systemic therapy including chemotherapy, external beam radiotherapy (EBRT)/intensity-modulated radiation therapy (IMRT), multikinase inhibitors (i.e., vandetanib, cabozantinib), selective RET inhibitors (i.e., pralsetinib, selpercatinib), or active surverillance (i.e., best supportive care), etc. To be mentioned, compared to the multikinase inhibitors which demonstrated limited efficacy on *RET*-driven cancers partially due to the off-target adverse effects, the next-generation highly potent selective RET inhibitors show improved efficacy and less toxicity. Two such drugs, pralsetinib and selpercatinib, have demonstrated remarkable clinical efficacy and safety in phase I/II trails (83, 84). Although pralsetinib has not yet been approved and would not be offered outside of a clinical trial for the moment, selpercatinib has been recently approved by the US FDA for the treatment for *RET*-mutant MTC (85). In addition, some other selective RET inhibitors, such as BOS172738, TPX-0046, and TAS0953/HM06, are in early stage of development (86). The mechanisms of acquired resistance to the multikinase inhibitors and selective RET inhibitors is an area of active research and secondary *RET* alterations (*RET* S904F, I788N, V804L/M, and G810A/S/R), acquired non-*RET* alterations (*MDM2* amplification and *NRAS* Q61K), and activation of bypass signaling (activation of MAPK, EGFR, and AXL) were known mechanisms involved (86). The optimal personalized treatment decision in recurrent/metastatic MTC ultimately depends on the balance between the rate of MTC progression and the quality of life without treatment and the efficacy and side effects of therapy (Figure 2) (2, 11, 36, 82, 87). The ATA-2015 recommended screening for PHEO/HPTH beginning at 11 and 16 years of age for individuals with ATA-H~HST mutations and ATH-MOD mutations, respectively (2). The screening method consists of measuring free plasma or fractionated urinary MN/NMN, serum calcium, and parathyroid hormone (PTH) levels, as well as CT or MRI imaging for patients with positive biochemical results (2). Laparoscopic or retroperitoneoscopic adrenalectomy should be performed after appropriate preoperative preparation for treatment of MEN2-related PHEO (2). However, patients can have significant risk for Addisonian-like complications and consequent lifelong dependency on steroids following bilateral adrenalectomy. Therefore, procedures that can minimize these complications are needed. Previous studies demonstrated that subtotal adrenalectomy (adrenal-sparing surgery, ASS) can offer sufficient adrenocortical stress capacity and in turn allow patients to avoid corticosteroid supplementation (88). A study by Scholten et al. (89) showed that, compared to unilateral total adrenalectomy, unilateral ASS has comparable recurrence rates and eventually fewer complications associated with steroid replacement, indicating that unilateral ASS is a feasible treatment option for MEN2-related PHEO. A recent international retrospective population-based study conducted by 30 academic medical centers (including our center) showed that, compared to total adrenalectomy, the rate of PHEO recurrence after ASS was comparable, whereas the risk of postoperative adrenal insufficiency and steroid dependency was significantly lower (87 vs. 43%; *P* = 0.03) (90). These results indicated that ASS is a highly successful treatment approach of choice that can reduce the frequency of complications and should be considered for all MEN2-related PHEO, although ASS was considered as an alternative procedure to adrenalectomy in the ATA-2015 guidelines (11, 90, 91). There are several issues that should be considered when selecting ASS as a treatment option (2, 11, 57, 90). First, the presence of a PHEO must be excluded prior to any MEN2-related surgical procedure. The ASS should be performed first to prevent hypertensive crisis or even death during other operations. Second, patients should receive adequate oral administration of alpha-blockers and active expansion therapy before the ASS procedure to reduce or avoid extreme perioperative blood pressure fluctuation. Third, for patients that have undergone bilateral ASS, perioperative glucocorticoid, and mineralocorticoid replacement is necessary to prevent Addisonian crisis. Fourth, due to the limited clinical data concerning MEN2B-related PHEO, most current treatment methods are based on treatment principles for MEN2A-related PHEO, and the preferred treatment option is laparoscopic ASS with the preservation of adrenocortical function. In contrast, clinically asymptomatic MEN2B-related PHEO can be detected and diagnosed early, as MEN2B patients are monitored more frequently. Meanwhile, MEN2B-related PHEO is not more progressive than MEN2A-related PHEO (4, 92). Of note, fertile MEN2 women are at high risk of complicated pregnancy because unrecognized PHEO that may lead to severe, and even fatal adverse maternal or fetal outcomes (93, 94). PHEO in female MEN2 patients should be treated 3 months prior to a planned pregnancy, and for patients who are already pregnant, PHEO should be treated before the gestational age of 28 weeks (2). More recently, one multicenter study was conducted by Bancos et al. (including our group) that focused on the presentation, management, and outcomes of women with PHEO during pregnancy. The results of this study indicated that both maternal and fetal outcomes were good, even if the PHEO was metastatic, and particularly when the PHEO was diagnosed before or during pregnancy. However, unrecognized and untreated PHEO was associated with a 27-fold higher risk of either maternal or fetal complications (*not published*). Thus, screening and exclusion of PHEO in female MEN2 patients who plan to become pregnant is critical.

With respect to treatment of MEN2A-related HPTH, only visibly enlarged parathyroid glands should be resected with intraoperative PTH monitoring to document complete removal of hyperfunctioning parathyroid tissue (2, 95). The surgical options for patients having enlargement of all four glands include subtotal parathyroidectomy with a piece of one gland left *in situ* on a vascular pedicle or total parathyroidectomy with a heterotopic autograft (2, 82).

ASS followed by TT and parathyroidectomy in a single procedure performed in experienced centers might also be a preferred surgical choice to treat coexisting MTC, PHEO, or HPTH. Such procedures have already been successfully performed (Table 4) (96–99). However, individualized hormone replacement/supplement and further clinical studies are still needed to confirm the effectiveness of this approach.

**Table 4:** Summary of five MEN2 patients with successive adrenalectomy, thyroidectomy, and parathyroidectomy in a single procedure.

**References**	**Age(years)/gender**	**MEN2 type**	***RET* mutation**	**Diagnosis**	**Surgical treatment**	**Postoperative outcome**
Spapen et al. (96)	37/F	MEN2A	–	MTC, bilateral PHEO, and HPTH	Bilateral adrenalectomies, TT, and PD	Right lung MTC metastasis 7 years after the initial surgery
Spinelli et al. (97)	–	MEN2A	–	Bilateral PHEO and HPTH	Bilateral laparoscopicadrenalectomies, preventive TT, and PTD	–
McIntyre et al. (98)	29/M	MEN2A	C634	MTC, bilateral PHEO, and HPTH	Bilateral adrenalectomies, TT + LND, and subtotal PTD	Uneventful recovery and no evidence of recurrence
Efared et al. (99)	40/F	MEN2A	C634R	MTC, left PHEO, right cPHEO/PGL, and HPPH	Bilateral adrenalectomies, TT + cervical LND, and PTD	Good recovery and no signs of recurrence 3 years post-operation
Our center, 2018	40/F	MEN2A	C634R	MTC, left PHEO, and HPTH	Left laparoscopic ASS, TT + cervical LND, and PTD	Recovery and no signs of recurrence 7 months post-operation

*M, Male; F, Female; MEN2, multiple endocrine neoplasia type 2; MTC, medullary thyroid carcinoma; PHEO, pheochromocytoma; HPTH, hyperparathyroidism; cPHEO/PGL, composite pheochromocytoma/paragangliomas; LNM, lymph node metastasis; PH, parathyroid hyperplasia; TT, total thyroidectomy; LND, lymph node dissection; PTD, parathyroidectomy; ASS, adrenal-sparing surgery*.

### Psychological Support and Participation

The diagnosis of MEN2 and subsequent on-going clinical care can have a negative impact on an individual's quality of life and psychological well-being (100–102). MEN2 patients would face many challenges, including fear for the future, decisions about having children, side effects of cancer treatment, coping behaviors in the face of a chronic and frequently incurable cancer, and difficulties in access to adequate health care (101). Worse health-related quality of life in all 7 domains, including anxiety, depression, fatigue, pain interference, physical functioning, sleep disturbance, and ability to participate in social roles, in the Patient-Reported Outcomes Measurement Information System were reported by MEN2 patients (103). Previous study shown that a good understanding about the disease and available treatments, together with the support of a reliable, experienced, and multidisciplinary medical team (i.e., Association for Multiple Endocrine Neoplasia Disorders, AMEND, https://www.amend.org.uk/), was associated with a substantial reduction in psychosomatic complaints (102, 104). Thus, psychological support and genetic counseling should be provided throughout the entire process, including *RET* gene testing for MEN2 patients and their family members and well as discussion of reproductive options, preconception and prenatal testing, clinical screening and early clinical interventions, and post-operative monitoring, to improve the quality of life of MEN2 patients (2).

During the whole process, full participation of physicians, patients and family members, government, scientific researchers, pharmaceutical companies, and non-governmental organizations which provide resources for the medical team that will help inform management (such as NORD: National Organization for Rare Disorders, https://rarediseases.org/; EURORDIS: The Voice of Rare Disease Patients in Europe, https://www.eurordis.org/; OSSE: Open Source Registry System for Rare Diseases, https://www.osse-register.de/en/; NRDRS: National Rare Diseases Registry System of China, https://www.nrdrs.org.cn/app/rare/index.html) are required to promote effective management of MEN2. The physicians should be trained to increase the knowledge of MEN2, such as optimal timing and extent of surgery, to improve the long-term disease-free survival rate of patients with MEN2. Patients and their families should be aware of the risk of MEN2 and the importance of prevention, early diagnosis and early normalized treatment from recognized patient support groups to avoid misleading information obtained from digital media. They should also be encouraged to follow intervention strategies as early as possible and the children in their families should receive age-appropriate information in a friendly and caring way. More efforts, such as strengthening MEN2 treatment infrastructure; promoting of *RET* screening and MEN2-related research programs (development of new molecular drugs, stem cell therapy, and gene theraphy following ethical principle); introducing relevant policies to provide subsidies or reimbursements for expensive costs of therapy; developing a more detailed MEN2 database (i.e., https://arup.utah.edu/database/MEN2/MEN2_welcome.php) including the information of diagosis, treatment, and follow-up of the patients worldwide based on full respect for the privacy of patients and their families, are also indispensable to effectively avoid and reduce the likelihood of unfavorable clinical outcomes due to MEN2.

## Conclusion

Over the past years, the gleaned insight into the natural course of disease caused a paradigm shift in the management of MEN2. Future research should delineate further the pathophysiology of this rare disease (i.e., the oncogenic signaling, the reasons for the intra-/inter- familial phenotypic variability, and the more precise mutation-specific and age-dependent penetrance of MTC/PHEO/HPTH) to promote the development more potent and specific therapeutic strategies (i.e., cancer vaccines and gene therapy). In summary, MEN2 can be managed using an approach that involves 5Ps: prevention, prediction, personalization, psychological support, and participation. The occurrence of MEN2 could be ***prevented***through the implementation of PD or PGT-M based on *RET* mutations. Identification of *RET* pathogenic mutations can enable early diagnosis of MEN2. By combining *RET* mutation testing with measurement of Ctn/MN/NMN/PTH levels, risk stratification, and progression of MEN2 could be accurately ***predictive***, thus facilitating implementation of ***personalized***precision treatments to improve disease-free survival and overall survival. Furthermore, MEN2 awareness needs to be improved, which requires ***participation***of physicians, patients, family members, and related organizations. P***sychological***support is also important to promote effective management of MEN2. The clinical utility of the “5P” strategies for MEN2, which represent a paradigm of precision medicine, could effectively improve the health of MEN2 patients, and ultimately eliminate the adverse outcomes of MEN2 (50, 105).

## Author Contributions

X-PQ conceived and carried out the 5P strategies. The manuscript was written and approved by X-PQ, S-YL, Y-QD, Y-LS, C-MX, and M-JY. All authors contributed to the article and approved the submitted version.

## Conflict of Interest

The authors declare that the research was conducted in the absence of any commercial or financial relationships that could be construed as a potential conflict of interest.
